# Metabolite profiling reveals new insights into the regulation of serum urate in humans

**DOI:** 10.1007/s11306-013-0565-2

**Published:** 2013-07-20

**Authors:** Eva Albrecht, Melanie Waldenberger, Jan Krumsiek, Anne M. Evans, Ulli Jeratsch, Michaela Breier, Jerzy Adamski, Wolfgang Koenig, Sonja Zeilinger, Christiane Fuchs, Norman Klopp, Fabian J. Theis, H.-Erich Wichmann, Karsten Suhre, Thomas Illig, Konstantin Strauch, Annette Peters, Christian Gieger, Gabi Kastenmüller, Angela Doering, Christa Meisinger

**Affiliations:** 1 Institute of Genetic Epidemiology, Helmholtz Zentrum München, German Research Center for Environmental Health, Ingolstädter Landstr. 1, 85764 Neuherberg, Germany; 2 Research Unit of Molecular Epidemiology, Helmholtz Zentrum München, German Research Center for Environmental Health, Neuherberg, Germany; 3 Institute of Bioinformatics and Systems Biology, Helmholtz Zentrum München, German Research Center for Environmental Health, Neuherberg, Germany; 4 Metabolon, Inc., 617 Davis Drive, Suite 400, Durham, NC 27713 USA; 5 Institute of Experimental Genetics, Genome Analysis Center, Helmholtz Zentrum München, German Research Center for Environmental Health, Neuherberg, Germany; 6 Lehrstuhl für Experimentelle Genetik, Technische Universität München, Munich, Germany; 7 Department of Internal Medicine II-Cardiology, University of Ulm Medical Center, Ulm, Germany; 8 Hanover Unified Biobank, Hanover Medical School, Hanover, Germany; 9 Institute of Epidemiology I, Helmholtz Zentrum München, German Research Center for Environmental Health, Neuherberg, Germany; 10 Institute of Medical Informatics, Biometry, and Epidemiology, Chair of Epidemiology, Ludwig-Maximilians-Universität, Munich, Germany; 11 Klinikum Grosshadern, Munich, Germany; 12 Department of Physiology and Biophysics, Weill Cornell Medical College in Qatar, Education City-Qatar Foundation, Doha, Qatar; 13 Institute of Medical Informatics, Biometry and Epidemiology, Chair of Genetic Epidemiology, Ludwig-Maximilians-Universität, Munich, Germany; 14 Institute of Epidemiology II, Helmholtz Zentrum München, German Research Center for Environmental Health, Neuherberg, Germany; 15 Munich Heart Alliance, Munich, Germany; 16 Central Hospital of Augsburg, Monitoring Trends and Determinants on Cardiovascular Diseases/Cooperative Research in the Region of Augsburg Myocardial Infarction Registry, Augsburg, Germany; 17 Member of German Center for Diabetes Research (DZD), Neuherberg, Germany; 18 Institute of Genetic Epidemiology, Helmholtz Zentrum München, German Research Center for Environmental Health, Neuherberg, Germany

**Keywords:** Gaussian Graphical Modeling, Metabolite network, Pathway reconstruction, Allopurinol, Uric acid, Purine metabolism

## Abstract

Serum urate, the final breakdown product of purine metabolism, is causally involved in the pathogenesis of gout, and implicated in cardiovascular disease and type 2 diabetes. Serum urate levels highly differ between men and women; however the underlying biological processes in its regulation are still not completely understood and are assumed to result from a complex interplay between genetic, environmental and lifestyle factors. In order to describe the metabolic vicinity of serum urate, we analyzed 355 metabolites in 1,764 individuals of the population-based KORA F4 study and constructed a metabolite network around serum urate using Gaussian Graphical Modeling in a hypothesis-free approach. We subsequently investigated the effect of sex and urate lowering medication on all 38 metabolites assigned to the network. Within the resulting network three main clusters could be detected around urate, including the well-known pathway of purine metabolism, as well as several dipeptides, a group of essential amino acids, and a group of steroids. Of the 38 assigned metabolites, 25 showed strong differences between sexes. Association with uricostatic medication intake was not only confined to purine metabolism but seen for seven metabolites within the network. Our findings highlight pathways that are important in the regulation of serum urate and suggest that dipeptides, amino acids, and steroid hormones are playing a role in its regulation. The findings might have an impact on the development of specific targets in the treatment and prevention of hyperuricemia.

**Electronic supplementary material:**

The online version of this article (doi:10.1007/s11306-013-0565-2) contains supplementary material, which is available to authorized users.

## Introduction

Since the early 1800s, hyperuricemia is known to be causally involved in the pathogenesis of gout, a painful inflammatory arthritis induced by the deposition of monosodium urate crystals in synovial fluid and other tissues (Kanbay et al. 2011; Neogi 2011). Increased serum urate concentrations are implicated in cardiovascular disease and elevated urate is associated with obesity, hypertension and insulin resistance, and consequently with the metabolic syndrome and type 2 diabetes (Hayden and Tyagi 2004; Koenig and Meisinger 2008). By contrast, the relatively high serum urate levels of humans compared to most other mammals are believed to play a positive role as an antioxidant (Wu et al. 2011). Thus, human physiology is especially sensitive to the precise range of serum urate levels.

In humans and higher primates, who have lost hepatic uricase activity during evolution, serum urate is the final oxidation product of purine metabolism. Serum urate is produced by xanthine oxidase from xanthine and hypoxanthine and is excreted in urine by the proximal tubular cells of the kidney. The regulation of serum urate concentrations is regarded as a result of complex interplays between genetic, lifestyle, and environmental factors. Recently, genome-wide association studies (GWAS) have identified single nucleotide polymorphisms (SNPs) associated with serum urate concentrations and gout, several of them located in genes coding for renal transport proteins (Doring et al. 2008; Kolz et al. 2009; Kottgen et al. 2013; Li et al. 2007; Vitart et al. 2008; Wallace et al. 2008; Yang et al. 2010). However, despite the success of these GWAS, detailed functional information on the underlying biological processes is still lacking.

Metabolomics, the study of ideally all metabolites in a biological system, is one of the youngest of the “omics” sciences. Metabolites are the end products of cellular regulatory processes and may provide more details on potentially affected biological pathways (Ma et al. 2012). The detection and functional characterization of such pathways is crucial to improve management and treatment of patients with hyperuricemia and gout.

In order to investigate the metabolic vicinity of serum urate, we performed metabolite profiling of 1,764 individuals of the KORA F4 survey and examined serum urate connected metabolites using Gaussian Graphical Models (GGMs). In previous studies, we have demonstrated these statistical models to reconstruct metabolic pathways from large-scale metabolomics data (Krumsiek et al. 2011). To address the pronounced sex differences in the regulation of serum urate concentrations we analyzed sex differences within the network. Additionally, we analyzed the influence of urate lowering medication for all metabolites within the generated network.

## Materials and methods

### Study population

The KORA studies (Cooperative Health Research in the Region of Augsburg) is a series of independent population based studies from the general population living in the region of Augsburg, southern Germany (Holle et al. 2005; Wichmann et al. 2005). The KORA S4 survey, was conducted in 1999–2001 including 4,261 participants (response 67 %). A total of 3,080 subjects participated in the follow-up examination KORA F4 in 2006–2008. The present analysis comprises 1,764 KORA F4 participants (908 females and 856 males) in an age range of 32–81 years (mean 60.86 years). Of those, 83 participants were treated by urate lowering medication (17 females and 66 males). All 83 were treated by allopurinol (uricostatic drug) and four of them additionally by benzbromaron (uricosuric drug). Written informed consent has been given by all participants and the study has been approved by the Ethics Committee of the Bavarian Medical Association.

### Blood sampling

Blood samples were collected as part of the KORA F4 follow-up. To avoid variation due to circadian rhythm, blood was drawn in the morning between 08:00 and 10:30 after a period of at least 10 h overnight fasting. Material was drawn into serum gel tubes, gently inverted twice and then allowed to rest for 30 min at room temperature (18–25 °C) to obtain complete coagulation. The material was then centrifuged for 10 min (2,750 g at 15 °C). Serum was divided into aliquots and kept for a maximum of 6 h at 4 °C, after which it was frozen at −80 °C until analysis.

### Metabolomics measurements

Metabolites were measured in 1,768 subjects from the KORA F4 study by Metabolon, Inc. (Durham, NC, USA), a commercial supplier of metabolic analyses, who has developed a platform that integrates the chemical analysis, including identification and relative quantification, data-reduction and quality-assurance using three separate analytical methods (GC–MS, LC–MS (positive mode), LC–MS (negative mode)) to detect as wide a range of metabolites as possible (Evans et al. 2009; Suhre et al. 2011).

Sample preparation was assisted by a Hamilton ML STAR (Hamilton Company, Salt Lake City, UT, USA) robotics system. After thawing, 400 μl of extraction solvent (i.e. methanol, containing recovery standards) was added to each 100 μl of serum samples in a 96 deep well plate format. Extraction was carried out by shaking for 2 min using a Geno/Grinder 2000 (Glen Mills Inc., Clifton, NJ, USA). After centrifugation the supernatant was split into four aliquots: two for LC/MS analysis (positive and negative electrospray ionization mode), one for GC/MS analysis and one reserve aliquot. Solvent was removed on a TurboVap (Zymark) and the samples were dried under vacuum overnight. For LC/MS pos. ion mode samples were reconstituted with 0.1 % formic acid, for neg. ion mode with 6.5 mM ammonium bicarbonate pH 8.0. Both reconstitution solvents contained also internal standards. The GC/MS aliquots were derivatized for 1 h at 60 °C with *N,O*-bistrimethylsilyl-trifluoroacetamide in a solvent mixture of acetonitrile:dichlormethane:cyclohexane (5:4:1), containing 5 % triethylamine and retention time markers.

LC/MS analysis was performed on a LTQ mass spectrometer (Thermo Fisher Scientific Inc., Waltham, MA, USA) equipped with a Waters Acquity UPLC system (Waters Corporation, Milford, MA, USA). Two separate columns (2.1 × 100 mm Waters BEH C18 1.7 μm particle) were used for acidic (solvent A: 0.1 % formic acid in H2O, solvent B: 0.1 % formic acid in methanol) and basic (A: 6.5 mM ammonium bicarbonate pH 8.0, B: 6.5 mM ammonium bicarbonate in 98 % methanol) mobile phase conditions, optimized for positive and negative electrospray ionization, respectively. After injection of the sample extracts the columns were developed in a gradient of 100 % A to 98 % B in 11 min runtime at 350 μl/min flow rate. The eluent flow was directly connected to the ESI source of the LTQ mass spectrometer. Full scan mass spectra (99–1000 *m*/*z*) and data dependent MS/MS scans with dynamic exclusion were recorded in turns.

GC/MS analysis was done on a Thermo-Finnigan Trace DSQ fast-scanning single-quadrupole mass spectrometer, equipped with a 20 m × 0.18 mm GC column with 0.18 μm film phase consisting of 5 % phenyldimethyl silicone. Electron impact ionization at 70 eV was used and the column temperature was ramped between 60 and 340 °C with helium as carrier gas. Mass spectra in a scan range from 50 to 750 *m*/*z*, were recorded.

Metabolites were identified from the LC/MS and GC/MS data by automated multiparametric comparison with a proprietary library, containing retention times, *m*/*z* ratios, and related adduct/fragment spectra for over 1,500 standard compounds measured by Metabolon. For each identified metabolite the raw area counts were normalized to the median value of the run day to correct for inter-day variation of the measurements.

The panel includes 517 untargeted metabolites, spanning several metabolic classes (amino acids, acylcarnitines, sphingomyelins, glycerophospholipids, carbohydrates, vitamins, lipids, nucleotides, peptides, xenobiotics and steroids). The quantified metabolites can be distinguished into chemically identified metabolites, and unidentified, here called “unknown” metabolites. Nine of those unknown metabolites have recently been identified by Krumsiek et al. (2012). Urate is one of the measured metabolites on the panel.

From the original data matrix containing 1,768 samples and 517 metabolites, we first excluded metabolites with more than 20 % missing values and then samples with more than 10 % missing values. The filtered data matrix contained *n* = 1,764 samples and 355 metabolites (241 known and 114 unknown). All normalized ion counts were transformed by natural logarithm and missing values were imputed using the ‘mice’ R package (van Buuren and Groothuis-Oudshoorn 2011). Detailed information about all analyzed metabolites is provided in Supplementary Table 1.

### Identification of the unknown metabolite X-11422

Within the generated GGM xanthine was not directly connected to urate and hypoxanthine but via the unknown metabolite X-11422 (see Supplementary Fig. 1). The central position of X-11422 in this well-known pathway induced speculations about its chemical identity. Following the ideas for unknown identification in Krumsiek et al. (2012), we defined possible candidates by considering the direct neighbors of X-11422 in the GGM, its mass, and its fragmentation spectrum: alloxanthine, the primary metabolite of allopurinol, or xanthine itself displaying altered chromatographic characteristics. Xanthine was originally measured and identified on two out of the three analytical methods used to profile metabolites. Interestingly, xanthine was not detected on the third platform; rather X-11422 was detected. In order to determine if the unknown could be either one of these candidates, we performed a co-elution spiking experiment. We spiked several candidate molecules, including xanthine and alloxanthine, that had the same molecular formula (as determined by accurate mass spectrometric analysis using an Orbitrap Elite mass spectrometer operated at 30,000 resolution) and fragmentation spectrum when run neat, into both urate medication treated and non-treated patient samples. Specifically, treated and non-treated medication samples were run with no spike, low, medium, and high spikes of both xanthine and alloxanthine separately, as well as spiked with both molecules simultaneously, to determine if either candidate co-eluted with the peak identified as X-11422. Positive confirmation of identity of X-11422, given the candidates already displayed identical molecular formula and fragmentation spectrum, would show an exact co-elution of the candidate molecules with the unknown in the treated samples.

This experiment demonstrated that xanthine, not alloxanthine, co-eluted perfectly in this matrix with the peak identified as X-11422 and therefore X-11422 represented an alternate measurement of xanthine. The original measurement of xanthine and the alternate measurement of xanthine (X-11422) correlate with a Pearson Correlation of r = 0.60 and show highly similar response to treatment. It is interesting to note that the alternate measurement of xanthine showed an increased correlation to the treatment as opposed to the original measurement. Since these two measurements are occurring on two different analytical methods with different background and chemical noise characteristics, slight differences in relative quantitation is not surprising.

### Medication ascertainment

All participants were asked to bring to the interview all medications taken in the 7 days preceding the examination. Medication data was obtained online using the IDOM program (online drug-database leaded medication assessment). The medications were categorized according to the Anatomical Therapeutical Chemical (ATC) classification index.

### Statistical analysis

Within the original data matrix of 355 metabolites, partial correlations were calculated between each metabolite pair conditioning on age, sex, all other metabolites as well as SNPs which showed a significant association with at least one of the 355 known or unknown metabolites as described before (Krumsiek et al. 2012). Correlations between two metabolites were considered to be significant at a significance level of 0.05 and a correction for multiple testing by the false discovery rate (FDR) (Benjamini and Hochberg 1995; Benjamini and Yekutieli 2001). Significant partial correlations were visualized in a network graph, referred to a Gaussian graphical model (GGM). In previous studies, we have demonstrated that GGMs are able to reconstruct metabolic pathways from large-scale metabolomics data (Krumsiek et al. 2012, 2011). Within the GGM each node presents a metabolite and nodes are connected by an edge if their partial correlation is significant. Here, we visualize the network in a 3-neighborhood around urate, which means that all metabolites are assigned to the network graph if they are connected to urate by three or less edges.

The initially generated network is visualized in Supplementary Fig. 1. As the unknown metabolite X-11422 was located at a central position within the known purine pathway, we investigated on its identification as described above and identified it to be an alternate measurement of xanthine. In order to avoid duplicated metabolites within the graph, we removed known duplicates from the data-matrix and regenerated the GGM in the remaining 353 metabolites. The FDR corrected significance level of 0.05 involved a *p* value cutoff of 4.34 × 10^−5^. All metabolites within the network were tested for their associations with sex and urate lowering medication using a linear model which was additionally adjusted for age. Effects were considered to be significant below a threshold of 6.6 × 10^−4^, which corresponds to a Bonferroni correction for 76 independent tests.

Furthermore, we tested all pairwise partial correlations within the set of 353 metabolites for sex differences. Partial correlation coefficients were calculated separately in males and females. In contrary to the overall coefficients, we did not adjust for the set of SNPs in order to ensure a reliable estimation within these reduced sample sets. The male- and female-specific partial correlation coefficients were compared using a Fisher test (Levy and Narula 1978).

To compare the 58 edges within the generated urate GGM, a Bonferroni corrected significance level of 0.05/58 = 8.6 × 10^−4^ was applied. To test differences between all possible pairs of 353 metabolites, a Bonferroni corrected significance level of 0.05/62,128 = 8.0 × 10^−7^ was applied.

## Results and discussion

**Fig. 1:**
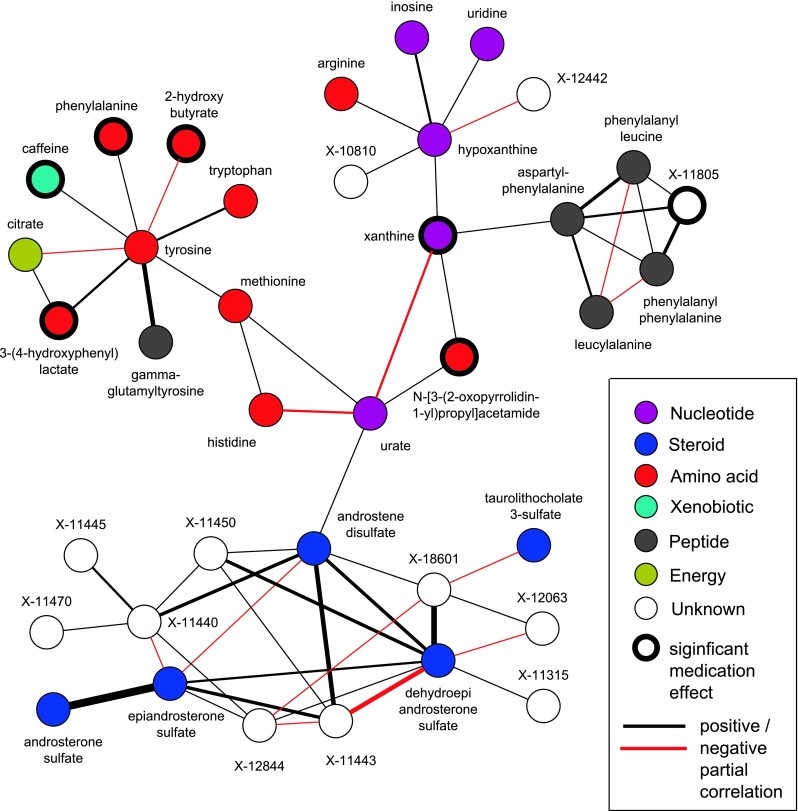
Serum urate GGM representing all significant associations within a three-neighborhood of serum urate. The thickness of each edge corresponds to the strength of partial correlation. Positive associations are marked as *black lines*, whereas negative correlations are represented by *red lines*. Metabolites are colored according to their biological pathways

In a hypothesis-free approach, we generated a data-driven GGM around serum urate, based on partial correlations. Thereby, 38 metabolites were assigned to a network, containing 26 known as well as 12 unknown metabolites (Fig. 1). Table 1 shows the corresponding partial correlation coefficients and *p* values for each of the edges within the network. The general structure of the serum urate network clusters into three parts of connected metabolites.

**Table 1:** Partial correlation coefficients for all significant associations within a 3-neighborhood of serum urate

Metabolite 1	Metabolite 2	Partial correlation coefficient	*p* value
Urate	Histidine	−0.231	9.08E−13
Urate	Methionine	0.181	2.54E−08
Urate	*N*-[3-(2-oxopyrrolidin-1-yl)propyl]acetamide	0.142	1.37E−05
Urate	Androstene disulfate	0.153	2.52E−06
Urate	Xanthine	−0.277	5.27E−18
Histidine	Methionine	0.188	7.06E−09
Methionine	Tyrosine	0.142	1.32E−05
Androstene disulfate	Dehydroepiandrosterone sulfate	0.351	1.40E−28
Androstene disulfate	Epiandrosterone sulfate	−0.176	5.38E−08
Androstene disulfate	X-18601	0.146	7.54E−06
Androstene disulfate	X-11440	0.352	1.13E−28
Androstene disulfate	X-11443	0.510	3.16E−63
Androstene disulfate	X-11450	0.156	1.60E−06
Xanthine	Aspartylphenylalanine	0.147	6.10E−06
Xanthine	Hypoxanthine	0.185	1.20E−08
Xanthine	*N*-[3-(2-oxopyrrolidin-1-yl)propyl]acetamide	0.140	1.68E−05
Tyrosine	2-hydroxybutyrate	−0.138	2.35E−05
Tyrosine	3-(4-hydroxyphenyl)lactate	0.319	1.37E−23
Tyrosine	Caffeine	0.136	2.91E−05
Tyrosine	Citrate	−0.138	2.24E−05
Tyrosine	Gamma-glutamyltyrosine	0.466	1.04E−51
Tyrosine	Phenylalanine	0.201	5.44E−10
Tyrosine	Tryptophan	0.268	7.02E−17
Dehydroepiandrosterone sulfate	Epiandrosterone sulfate	0.299	8.64E−21
Dehydroepiandrosterone sulfate	X-18601	0.574	3.66E−83
Dehydroepiandrosterone sulfate	X-11315	0.141	1.47E−05
Dehydroepiandrosterone sulfate	X-11443	−0.470	1.32E−52
Dehydroepiandrosterone sulfate	X-11450	0.392	8.38E−36
Dehydroepiandrosterone sulfate	X-12063	−0.193	2.51E−09
Dehydroepiandrosterone sulfate	X-12844	0.138	2.10E−05
Epiandrosterone sulfate	Androsterone sulfate	0.754	1.33E−172
Epiandrosterone sulfate	X-11440	−0.178	3.93E−08
Epiandrosterone sulfate	X-11443	0.411	1.48E−39
Epiandrosterone sulfate	X-12844	0.161	6.94E−07
X-18601	Taurolithocholate 3-sulfate	−0.134	4.04E−05
X-18601	X-12063	0.208	1.28E−10
X-18601	X-12844	−0.162	6.47E−07
X-11440	X-11445	0.314	7.74E−23
X-11440	X-11450	0.150	4.05E−06
X-11440	X-11470	0.140	1.75E−05
X-11440	X-12844	0.192	3.03E−09
X-11443	X-11450	0.213	4.88E−11
X-11443	X-12844	−0.155	2.00E−06
Aspartylphenylalanine	X-11805	0.256	1.85E−15
Aspartylphenylalanine	Leucylalanine	0.308	4.78E−22
Aspartylphenylalanine	Phenylalanylleucine	0.393	5.02E−36
Aspartylphenylalanine	Phenylalanylphenylalanine	0.155	1.94E−06
Hypoxanthine	Arginine	0.137	2.45E−05
Hypoxanthine	Inosine	0.254	3.05E−15
Hypoxanthine	Uridine	0.152	2.80E−06
Hypoxanthine	X-10810	0.162	6.03E−07
Hypoxanthine	X-12442	−0.140	1.80E−05
3-(4-hydroxyphenyl)lactate	Citrate	0.142	1.28E−05
X-11805	Phenylalanylleucine	0.142	1.36E−05
X-11805	Phenylalanylphenylalanine	0.342	4.55E−27
Leucylalanine	Phenylalanylleucine	−0.143	1.04E−05
Leucylalanine	Phenylalanylphenylalanine	−0.164	4.17E−07
Phenylalanylleucine	Phenylalanylphenylalanine	0.155	1.86E−06

The first cluster contains mainly nucleotides, namely xanthine, hypoxanthine, inosine, and uridine, as well as arginine, and two unknown metabolites. It includes the well-known pathway from inosine via hypoxanthine and xanthine to urate. Xanthine is further connected to a cluster of dipeptides, namely aspartyl-phenylalanine (aspartame), leucylalanine, phenylalanylphenylalanine, and phenylalanylleucine and one unknown metabolite X-11805. The conversion of hypoxanthine to xanthine and xanthine to urate is catalyzed by the rate-limiting enzyme xanthine oxidase, the only enzyme capable of catalyzing the formation of urate in man (Pfeffer et al. 1994). The complex mechanism by which xanthine oxidase catalyzes hypoxanthine and xanthine conversion has been described previously (Berry and Hare 2004; Hille and Massey 1981). Xanthine oxidase is significantly elevated in a variety of cardiovascular conditions such as coronary artery disease and heart failure (George and Struthers 2009). There is a large variability in human xanthine oxidase expression, which can be up to three-fold and on average 20 % higher in men than in women (Guerciolini et al. 1991). Although basal expression of xanthine oxidase is low in humans, hypoxias, IL-1, IL-6, TNF-α, lipo-polysaccharides as well as steroid treatment have been shown to up-regulate transcription (Berry and Hare 2004).

Aspartyl-phenylalanine (aspartame), a low-calorie sweetener, is directly connected to xanthine in the network. Aspartame was shown to pose antipyretic, analgesic and anti-inflammatory action and to delay osteoarthritis in animal models (LaBuda and Fuchs 2001; Manion et al. 2011; Pradhan et al. 2011). Interference of aspartame with rheumatoid factor activity has been proposed to alleviate the pain and immobility resulting from chronic inflammation of joints (Ramsland et al. 1999). Other (tryptophan-containing) dipeptides were shown to inhibit xanthine oxidase (Nongonierma and Fitzgerald 2012). The dipeptides connected to xanthine may therefore be interesting targets for the identification of novel treatments or prevention strategies for hyperuricemia and related diseases.

Urate and xanthine are further connected to *N*-[3-(2-oxopyrrolidin-1-yl)propyl]acetamide (acisoga), a metabolite of spermidine. Our metabolite network does not provide the first link between urate and spermidine. Spermidine and spermine were previously found to bind the organic anion transporter OAT1 in mice, and to be putative novel endogenous substrates of OAT1 (Ahn et al. 2011), which is also known to be a urate transporter (Ichida et al. 2003).

The second large cluster contains several essential amino acids and is connected via methionine and histidine to serum urate in our network. Histidine, tryptophan, and tyrosine are amino acids which are especially sensitive to hydroxyl radical exposure (Davies et al. 1987). Methionine enriched diet is known to decrease urate levels in chickens and ducks, whereas only a few small studies have analyzed the effect in humans (Xie et al. 2004). Furthermore, methionine can be demethylated to homocysteine and elevated homocysteine levels have, as well as elevated urate levels, been shown being a risk factor for atherosclerosis, coronary heart disease, and chronic kidney disease (Francis et al. 2004; Humphrey et al. 2008; Lubomirova et al. 2007). Significant associations between serum urate and homocysteine have been shown in plasma and serum (Lussier-Cacan et al. 1996; Malinow et al. 1995).

The third cluster correlated with serum urate is composed of steroids and several unknowns. The different concentrations of serum urate in both sexes and the higher incidence of gout in men compared to women, suggest a hormonal influence on the pathogenesis of gout (Gregolini et al. 1983). Excretion of urinary dehydroepiandrosterone and androsterone has been reported to be significantly lower in subjects with gout (Sparagana and Phillips 1972). A small study investigating the hormonal urinary excretion reported that patients previously treated with allopurinol showed slightly higher values of androsterone and dehydroepiandrosterone, and slightly lower values of 11-hydroxyandrosterone in comparison to normal subjects, suggesting different hormonal patterns between individuals with and without gout (Gregolini et al. 1983).

For all metabolites within the network we tested the influence of sex and urate lowering medication within a linear model. Table 2 shows the corresponding effect estimates. 25 of the 38 metabolites show strong differences between men and women (7.5 × 10^−5^ ≥ *p* ≥ 8.1 × 10^−196^). These large differences are in line with our previous work on sexual dimorphisms revealing significant concentration differences between males and females for 102 out of 131 metabolites (Mittelstrass et al. 2011).

**Table 2:** Influence of sex and urate lowering medication on levels of all metabolites within the 3-neighborhood of serum urate

Metabolite	Beta sex	*p* value sex	Beta medication	*p* value medication
2-Hydroxybutyrate	−0.108	1.16E−08	−0.169	1.75E−04
3-(4-Hydroxyphenyl)lactate	−0.340	1.81E−90	−0.175	4.10E−06
Androstene disulfate	−0.817	8.78E−103	−0.226	7.40E−03
Androsterone sulfate	−0.360	7.86E−23	0.092	2.84E−01
Arginine	0.019	1.05E−01	0.056	4.03E−02
Aspartylphenylalanine	−0.034	2.68E−01	−0.169	2.16E−02
Caffeine	−0.074	1.28E−01	−0.605	2.17E−07
Citrate	0.029	2.14E−02	−0.055	7.26E−02
Dehydroepiandrosterone sulfate	−0.436	1.85E−49	0.195	4.08E−03
Epiandrosterone sulfate	−0.518	6.28E−59	0.141	5.36E−02
Gamma-glutamyltyrosine	−0.091	2.71E−16	−0.076	3.53E−03
Histidine	0.032	5.29E−07	0.006	6.79E−01
Hypoxanthine	0.056	7.45E−05	−0.100	2.75E−03
Inosine	0.226	4.74E−09	0.034	7.12E−01
Leucylalanine	0.130	8.71E−08	0.117	4.12E−02
Methionine	−0.110	3.91E−52	0.054	1.35E−03
N-[3-(2-oxopyrrolidin-1-yl)propyl]acetamide	−0.026	1.11E−01	−0.275	3.46E−12
Phenylalanine	−0.056	1.69E−19	−0.076	1.92E−07
Phenylalanylleucine	−0.086	1.56E−03	−0.177	6.08E−03
Phenylalanylphenylalanine	−0.004	8.67E−01	−0.107	4.43E−02
Taurolithocholate 3-sulfate	−0.002	9.61E−01	−0.249	2.94E−03
Tryptophan	−0.080	2.99E−32	−0.036	2.16E−02
Tyrosine	−0.060	3.37E−12	−0.041	4.07E−02
Urate	−0.206	1.20E−112	0.027	1.78E−01
Uridine	0.009	3.42E−01	0.054	1.95E−02
Xanthine	0.059	3.66E−06	−0.896	7.08E−157
X-10810	−0.061	3.60E−03	0.058	2.43E−01
X-11315	0.151	1.40E−13	0.048	3.17E−01
X-11440	−0.600	3.04E−90	−0.069	3.03E−01
X-11443	−1.247	8.12E−196	−0.166	5.58E−02
X-11445	−0.081	1.32E−02	0.019	8.09E−01
X-11450	−0.512	1.61E−88	−0.049	3.97E−01
X-11470	−0.158	1.33E−17	0.102	1.94E−02
X-11805	−0.044	1.92E−01	−0.363	6.51E−06
X-12063	−0.207	1.14E−12	−0.207	2.69E−03
X-12442	0.126	8.04E−08	−0.093	9.24E−02
X-12844	0.030	1.01E−01	0.087	4.39E−02
X-18601	−0.550	2.13E−71	0.134	5.55E−02

The linear model was additionally adjusted for age (effects not shown). Males are coded 0, females are coded 1. Medication intake was coded with 1 compared to no medication intake 0

While the metabolites within the network expectedly show strong sex differences, we observe that the network itself is not sensitive to sex differences. To address this we compared all partial correlations within the network between men and women (Supplementary Table 2). Only eight of the 58 edges show a significant difference between men and women at a significance level of 8.6 × 10^−4^. Furthermore, we compared all pairwise partial correlations within the whole dataset of 353 metabolites. This global comparison shows that there are three edges below the significance level of 8.0 × 10^−7^ proving that there are no strong sex differences, which means that the network itself is not sex dependent.

**Fig. 2:**
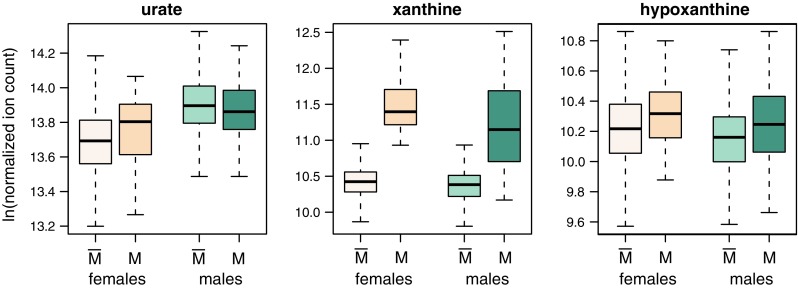
Levels of urate, xanthine, and hypoxanthine stratified between females and males as well as stratified between $\bar{\text{M}}$ = medication-free and M = medicated individuals. Medication-free females: *n* = 891, medicated females: *n* = 17, medication-free males: *n* = 790, and medicated males: *n* = 66)

Seven of the metabolites show a significant influence of urate lowering medication. The strongest influence of medication intake is seen for xanthine (*p* = 7.1 × 10^−157^). Furthermore, medication shows a significant influence on phenylalanine (*p* = 1.9 × 10^−7^), caffeine (*p* = 2.2 × 10^−7^), 3-(4-hydroxyphenyl)lactate (*p* = 4.1 × 10^−6^), 2-hydroxybutyrate (*p* = 1.7 × 10^−4^), *N*-[3-(2-oxopyrrolidin-1-yl)propyl]acetamide (*p* = 3.5 × 10^−12^), and X-11805 (*p* = 6.5 × 10^−6^). Allopurinol intake inhibits the enzyme xanthine oxidase which is responsible for the successive oxidation of hypoxanthine to xanthine and xanthine to urate. Figure 2 visualizes the medication and sex effects for urate, xanthine, and hypoxanthine. Urate levels themselves do not show differences between medicated and medication-free individuals (*p* = 0.18) and also for hypoxanthine the influence of medication is much weaker than on xanthine and not significant after correcting for multiple testing (*p* = 2.7 × 10^−3^). While the strong association between allopurinol intake and xanthine was expected, we additionally observed a medication influence on several amino acids, one unknown metabolite, and caffeine. Previous epidemiological studies found that coffee consumption is inversely associated with serum urate levels (Choi and Curhan 2007) and an influence of allopurinol medication on caffeine has been described (Birkett et al. 1997; Fuchs et al. 1999). Data on nutrition was not available, therefore we cannot exclude the possibility that the observed associations might be confounded by diet factors especially by those typically recommended for patients with gout.

## Concluding remarks

Metabolomic research has been expanded to epidemiological studies with the advent of new high-throughput technologies. Because of their immediacy to cell physiology, the measurement of metabolites provides a promising technique to discover new disease markers and pathways. We have generated a data-driven metabolite network around serum urate based on metabolite profiling of 1,764 individuals of the KORA F4 survey, to reconstruct pathways of biochemically related metabolites in a hypothesis-free approach.

We linked the regulation of serum urate to three different clusters of metabolites: While the connection to purine metabolism is well known, our current approach also links it to several essential amino acids and steroid hormones. We see sex differences for 25 of the 38 metabolites within the network. Furthermore, metabolites showed a dependency on uricostatic medication. Our findings may advert to new regulatory pathways and molecular mechanisms. This opens up new avenues for the identification of novel treatment targets and the prevention of hyperuricemia and related diseases as gout, cardiovascular disease and type 2 diabetes.

## Electronic supplementary material

Below is the link to the electronic supplementary material.

Supplementary material 1. Serum urate GGM representing all significant associations within a 3-neighborhood of serum urate. The GGM was generated on all available 355 metabolites, before the identification of the unknown metabolite X-11422 and the exclusion of duplicate metabolites. The thickness of each edge corresponds to the strength of partial correlation. Positive associations are marked as black lines, whereas negative correlations are represented by red lines. Metabolites are colored according to their biological pathways (EPS 479 kb)

Supplementary material 2 (XLSX 44 kb)

Supplementary material 3 (XLSX 20 kb)
